# From malaria to cancer: Computational drug repositioning of amodiaquine using PLIP interaction patterns

**DOI:** 10.1038/s41598-017-11924-4

**Published:** 2017-09-12

**Authors:** Sebastian Salentin, Melissa F. Adasme, Jörg C. Heinrich, V. Joachim Haupt, Simone Daminelli, Yixin Zhang, Michael Schroeder

**Affiliations:** 1 Biotechnology Center (BIOTEC), Technische Universität Dresden, 01307 Dresden, Germany; 2 B CUBE, Center for Molecular Bioengineering, Technische Universität Dresden, 01307 Dresden, Germany

## Abstract

Drug repositioning identifies new indications for known drugs. Here we report repositioning of the malaria drug amodiaquine as a potential anti-cancer agent. While most repositioning efforts emerge through serendipity, we have devised a computational approach, which exploits interaction patterns shared between compounds. As a test case, we took the anti-viral drug brivudine (BVDU), which also has anti-cancer activity, and defined ten interaction patterns using our tool PLIP. These patterns characterise BVDU’s interaction with its target s. Using PLIP we performed an in silico screen of all structural data currently available and identified the FDA approved malaria drug amodiaquine as a promising repositioning candidate. We validated our prediction by showing that amodiaquine suppresses chemoresistance in a multiple myeloma cancer cell line by inhibiting the chaperone function of the cancer target Hsp27. This work proves that PLIP interaction patterns are viable tools for computational repositioning and can provide search query information from a given drug and its target to identify structurally unrelated candidates, including drugs approved by the FDA, with a known safety and pharmacology profile. This approach has the potential to reduce costs and risks in drug development by predicting novel indications for known drugs and drug candidates.

## Introduction

Pharmaceutical companies spend around $ 2.6 billion in developing a drug through to market approval^1^. To minimize risk and development time, drug repositioning moves experimental or approved drugs to new indications, so that data from previously conducted safety and pharmacology studies can be leveraged. There are several examples of repositioning success stories, such as sildenafil (Viagra), which was originally developed for heart disease and was repurposed for erectile dysfunction, the sedative thalidomide, which is now approved for treatment of multiple myeloma and leprosy^2^, or the cytotoxic anti-cancer agent gemcitabine, which was originally developed as an anti-viral. The link between anti-viral and anti-cancer effects is also demonstrated with the small molecule brivudine (BVDU), which is approved for treatment of herpes and which has been investigated for use in pancreatic cancer^3^.

It appears surprising that one drug should be a cure for two diseases. One explanation is that a drug can bind promiscuously, i.e. to multiple different targets. In previous work, we established that drug promiscuity correlates with shared binding sites across the drug’s multiple targets^4^. Thus, structural analyses of shared binding sites and drug-target interactions are promising approaches to drug repositioning.

**Figure 1:**
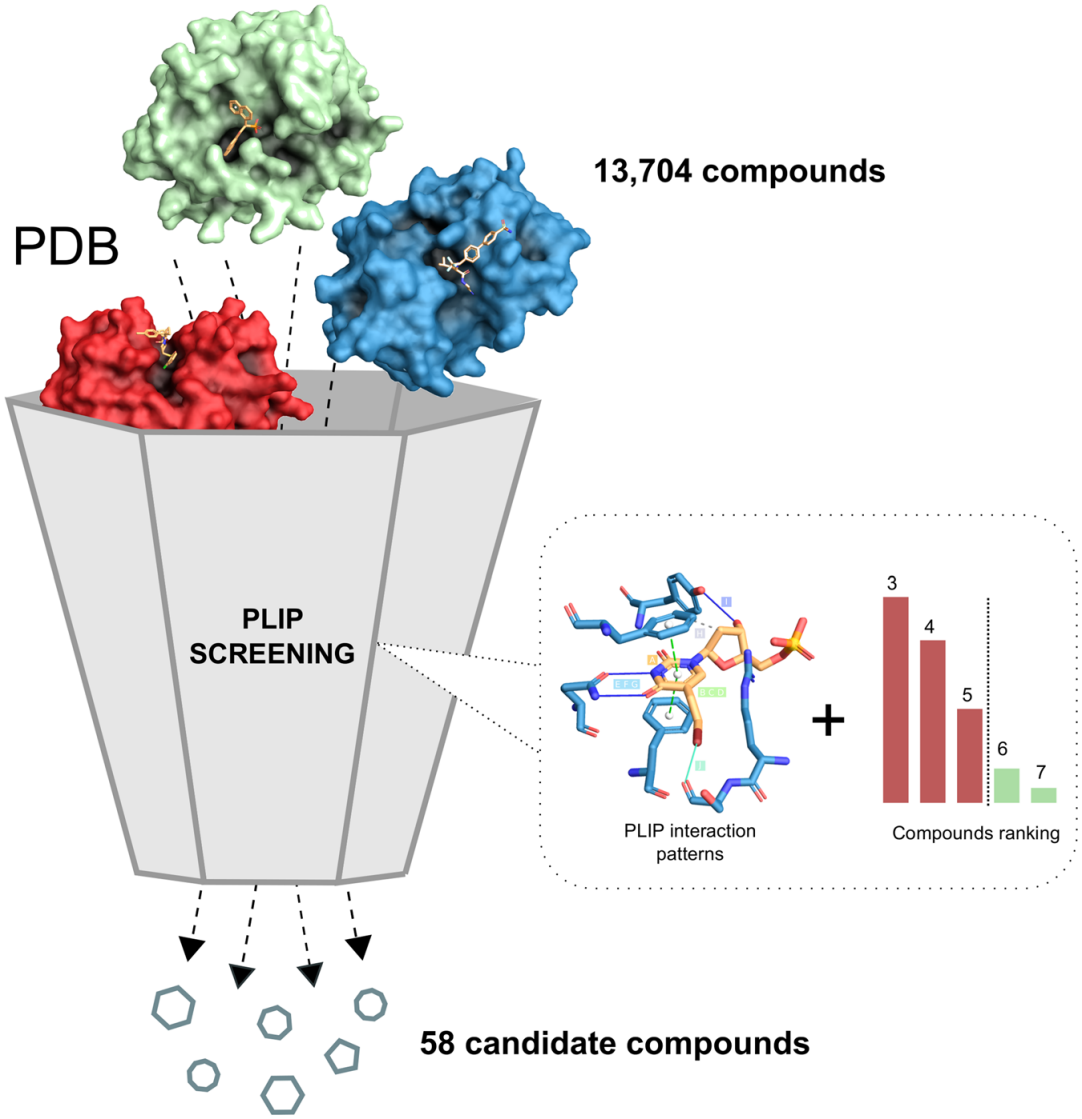
PLIP Screening. Screening for BVDU interaction patterns reduces an initial set with 13,704 compounds to 58 candidate compounds.

Such analyses hinge on the availability of structural data. While structural data is not as abundant as sequence data, it is growing steadily. The Protein Data Bank (PDB) has more than doubled in size in the last seven years. Today, it contains 3D structures of over 1,200 different drug targets^5^ and more than 60% of all PDB structures contain proteins complexed with biologically relevant ligands^4^. The growing availability of data is complemented by an increasing number of tools and methods mostly focusing on binding pockets^6–8^ or ligands^9–11^. A third approach characterises the interaction of ligands and binding pockets. Here, we recently introduced the Protein-Ligand Interaction Profiler (PLIP)^12^, a tool for comprehensive detection of molecular contacts. In this paper, we show how an analysis of a known drug-target interaction with PLIP can define interaction patterns, which can then be run against the PDB (Fig. 1). Since the patterns are structure-invariant, the screen against PDB will reveal ligands with novel scaffolds and novel targets. To document the power of this novel *in silico* screening approach, we tested it on the herpes drug BVDU. After defining the patterns through which BVDU interacts with its target proteins, we screened for compounds matching these patterns and then tested the hits *in vitro* for their potency in inducing cell killing in cultured cancer cells and inhibiting the function of a drug resistance target (anti-cancer effect).

## Results

In the 1980s, BVDU was first introduced as a treatment for *Herpes zoster* infection. BVDU is a thymidine analogue, composed of a nucleobase ring, a deoxyribose moiety, and a bromovinyl residue. In the infected cell, BVDU is phosphorylated by a viral thymidine kinase and then erroneously integrated into the viral genome^13^, terminating the viruses ability to replicate.

### BVDU interaction patterns for PLIP screening

**Figure 2:**
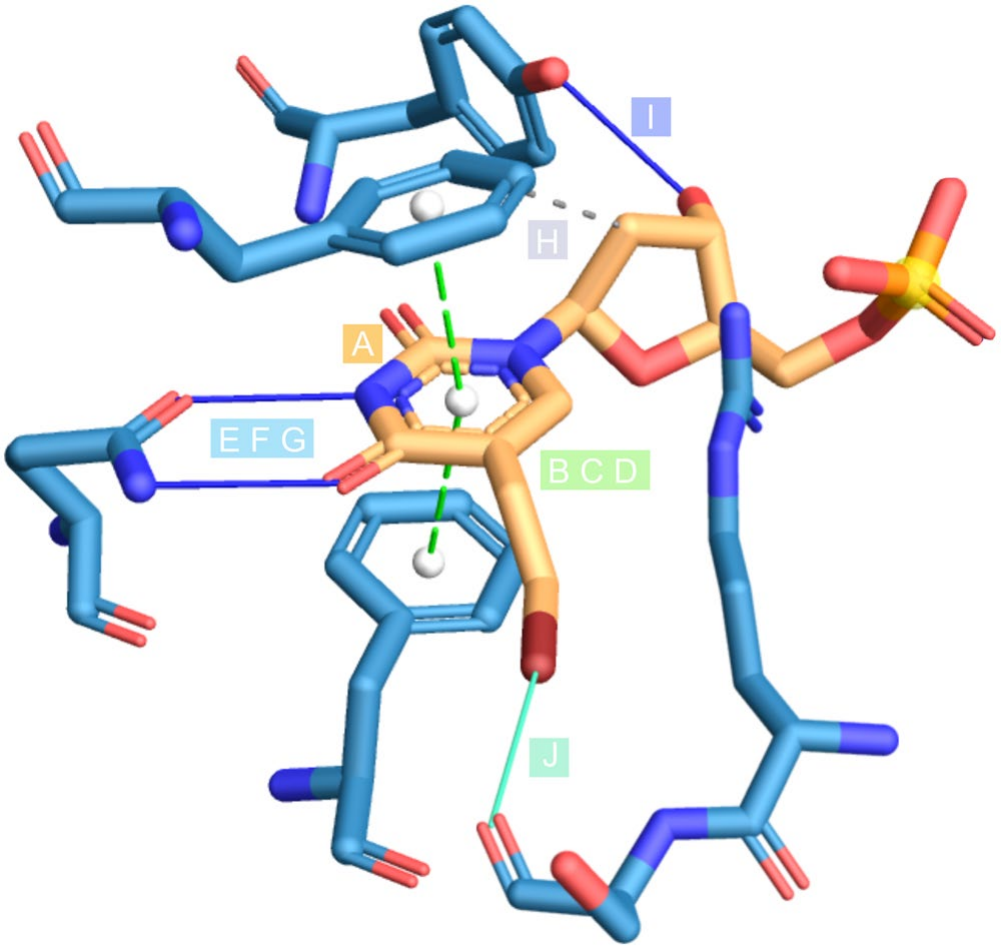
BVDU interaction patterns. Aromatic ring for *π*-stacking (**A**), double *π*-stacking to base ring (**B**), on opposite sites (**C**), in parallel (**D**), double hydrogen bonds to base ring (**E**), to the same residue (**F**), in parallel (**G**), distal hydrophobic contact (**H**), distal hydrogen bond (**I**) and distal halogen bond (**J**).

For our approach to structural drug repositioning it is important that crystal structure data for BVDU in complex with viral and non-viral kinases^14^ is available in PDB. Figure 2 shows in detail, how BVDU interacts with these kinases: BVDU’s nucleobase ring (A) engages in *π*-stacking (B–D) and features two prominent parallel hydrogen bonds (E–G). Its deoxyribose moiety has a hydrophobic contact (H) and a distal hydrogen bond (I) and the bromovinyl residue forms a halogen bond (J).

**Figure 3:**
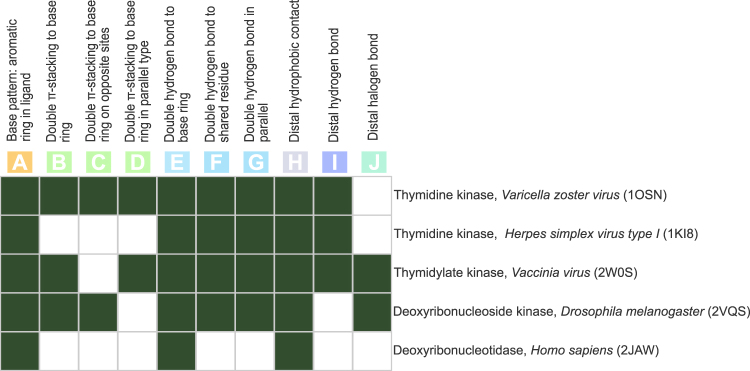
BVDU interactions patterns from five target proteins. Ten patterns relating to *π*-stacking (**A**–**D**), parallel hydrogen bonds (**E**–**G**), hydrophobic contacts (**H**), hydrogen bond (**I**) and a halogen bond (**J**) and their presence in five targets from five species.

The ten interaction patterns (A–J) were derived from five different PDB structures originating from viruses, human, and fruitfly. Given the variety of species it can be expected that not all patterns are present in all complexes. Even within the same species, there might be different use of the patterns, since not all interactions may be essential. Figure 3 demonstrates this point by showing presence and absence of the patterns in five PDB structures. A single *π*-stacking to BVDU’s aromatic ring (A), double hydrogen bonds to the base ring (E) and the distal hydrophobic contact (H) are present in all structures, whereas the parallel hydrogen bonds (F, G) are present in nearly all, and the double *π*-stacking patterns (B–D) only in some structures. Interestingly, the halogen bond (J) is not present in all viral structures. Overall, analysis with PLIP reveals some distinct features and combinations among the ten patterns, and we screened the PDB for these ten patterns, ranking compounds by the number of matches.

### PLIP screening of PDB for BVDU interaction patterns

**Figure 4:**
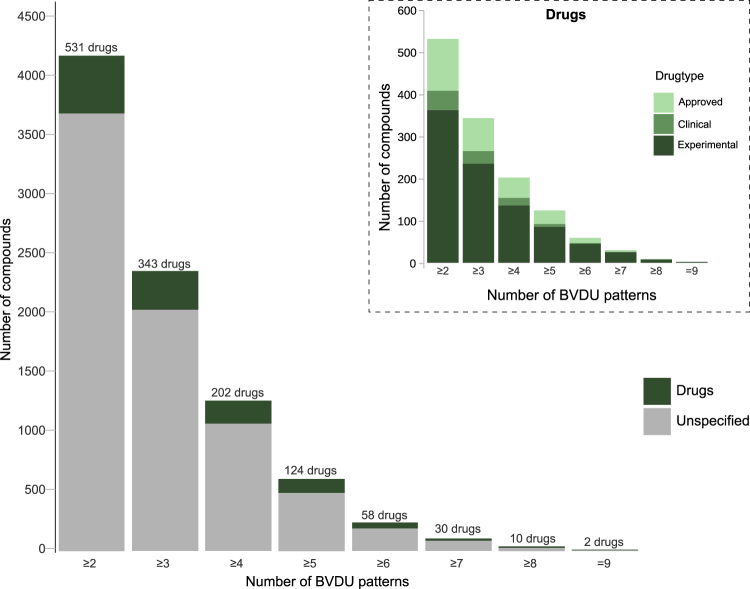
BVDU patterns effectively identify repositioning candidates. There are 247 compounds with six or more BVDU patterns including 12 approved drugs. One such drug is amodiaquine, an anti-malaria agent. As a control, thymidine, a BVDU analogue, satisfies nine BVDU patterns.

Our goal was to identify compounds which can form interactions in a similar manner to BVDU. We postulated that since these new compounds share interaction patterns with BVDU, they might also bind to the same target protein in cancer as BVDU. Screening the entire PDB for any ligands and targets, which interact similarly to BVDU, is not a trivial task as PDB comprises some 107,000 structures. After removing biologically irrelevant entries with BioLiP (see Methods), there are 170,219 complexes between 16,460 unique targets and 13,704 unique ligands. Each of these 170,219 complexes was analysed for the presence of the ten BVDU patterns and the results are shown in Fig. 4. There is no complex which contains all ten interaction patterns, but thymidine, the parent scaffold from which BVDU is derived, is among six ligands that contain nine patterns. Compounds were ranked by the number of patterns and annotated whether or not they were approved for therapy by the FDA. There are just 951 complexes containing six or more patterns and these complexes contain fewer than 250 compounds including 12 approved and 46 candidate drugs (i.e. experimental drugs or drugs in currently tested in clinical trials).

### Prioritization of repurposing candidates

Three criteria were used to prioritize candidate compounds: Firstly, we sought compounds with a different scaffold to BVDU. Secondly, we used FDA-approval to prioritise the results, since these compounds will have been extensively researched and information on safety pharmacology, dosing and formulation may be available. Such information can significantly increase the value of potential repositioning candidates. Thirdly, we sought an improvement in potency over BVDU, meaning the drug should show a stronger inhibition of BVDU’s target in cancer than BVDU itself.

**Figure 5:**
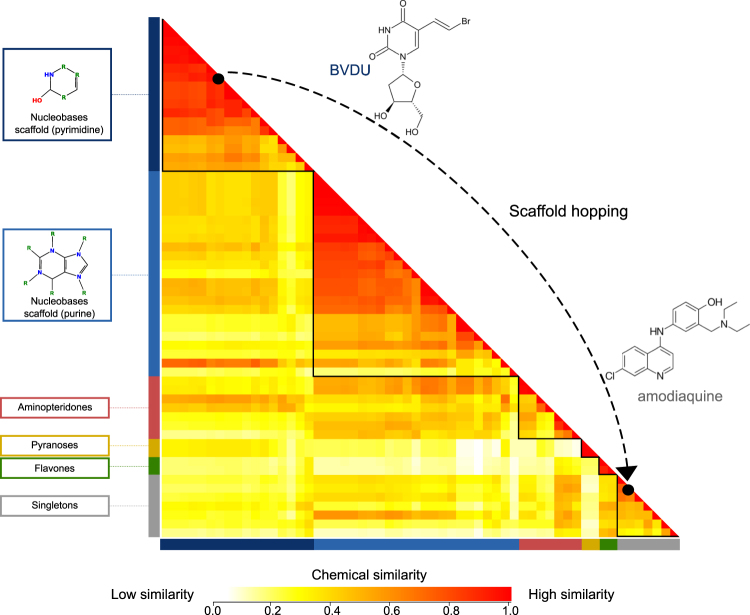
Scaffold hopping from BVDU to amodiaquine. Pairwise chemical similarity of compounds (red = similar, yellow = dissimilar) arranged by chemical scaffold and clustered by chemical similarity within each group. The top group shows BVDU among 40 nucleobases. Amodiaquine is one of 18 compounds with a scaffold different from BVDU.

Regarding scaffold diversity, we expected many of the top-scoring hit compounds to be nucleobases, since BVDU is a thymidine analogue. As mentioned above, thymidine is ranked highest with nine matching patterns and thus serves as a positive control. Similarly, deoxy-adenosine, -guanosine, and -cytidine contain seven BVDU patterns and deoxy-uridine contains six patterns. Overall, the 58 drugs with six or more patterns contain 40 such nucleobases. In Fig. 5 the 58 drugs are grouped according to chemical similarity and this reveals two large clusters (red boxes), which comprise pyrimidine and purine scaffolds. The remaining groups are much smaller in size and comprise scaffolds different from BVDU. These 18 novel compounds are of particular interest.

**Figure 6:**
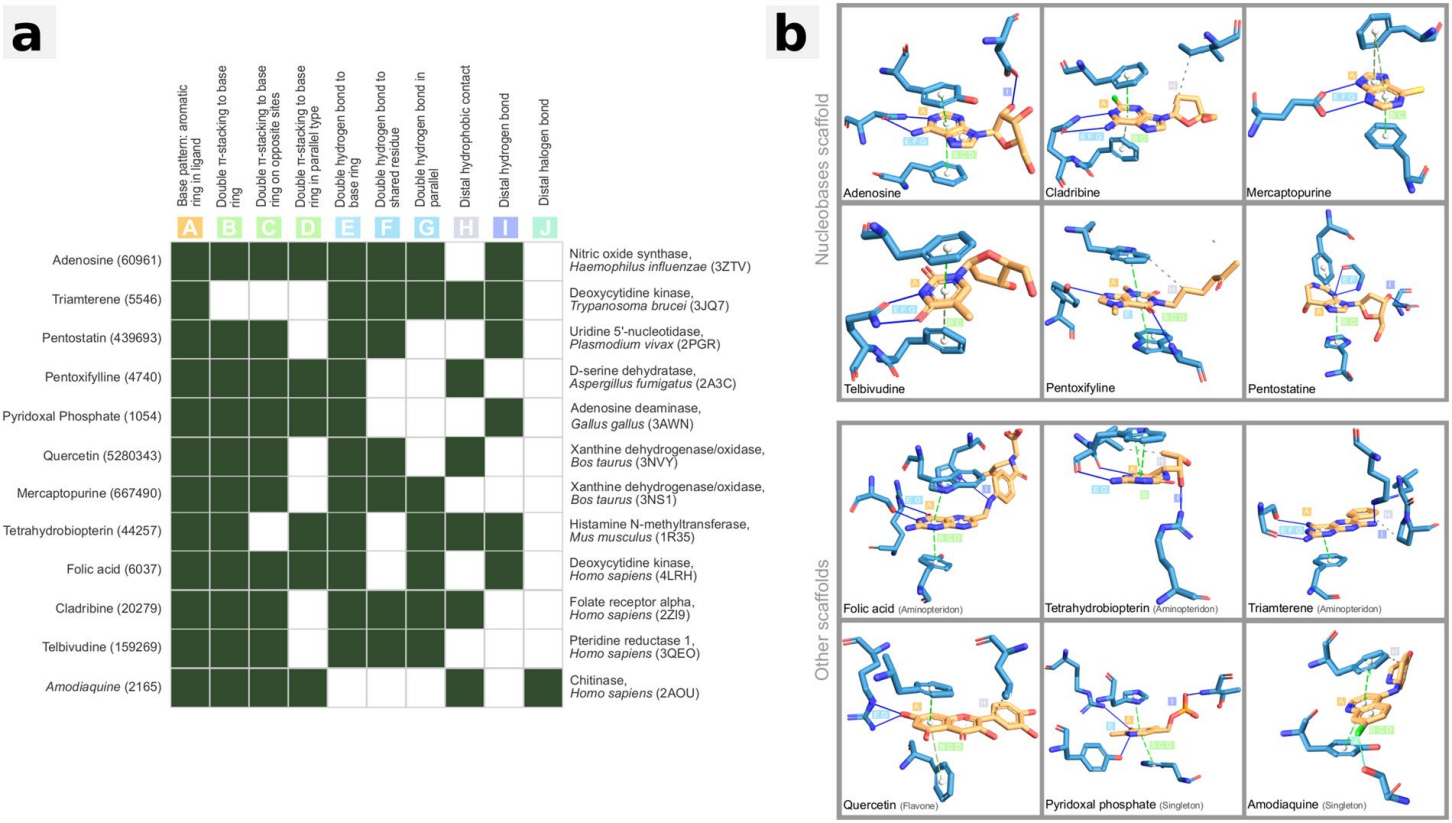
Approved drugs with six or more patterns. Twelve FDA-approved drugs across very different diseases and targets (panel A). Six are nucleobases like BVDU, but six are novel scaffolds. The patterns for *π*-stacking (**A**–**D**) and double hydrogen bond (**E**–**G**) are present in the majority of these compounds.

The second priorization aspect is FDA-approval: Among the 58 drugs, 12 are FDA-approved drugs which contain between 6 and 8 BVDU patterns (see Fig. 6 and Table 1). Half of these 12 FDA-approved drugs have a different scaffold to BVDU, namely the aminopteridones (folic acid, tetrahydrobiopterin, triamterene), the flavone quercetin as well as the singletons pyridoxal phosphate and amodiaquine.

**Table 1:** Approved drugs with six or more patterns.

Drug name	Target	Disease	Scaffold	N Patterns
Adenosine	Uridine 5′-nucleotidase	Myocardial perfusion scintigraphy and help identify coronary artery disease.	Nucleobases	8
Cladribine	Deoxycytidine kinase	Hairy cell leukemia	Nucleobases	7
Folic acid	Folate receptor alpha	Folate deficiencies and megaloblastic anemia.	Aminopteridones	7
Tetrahydrobiopterin	Nitric oxide synthase	Phenylketonuria(PKU)	Aminopteridones	7
Amodiaquine	Histamine N-methyltransferase	Acute malaria attacks in non-immune subjects	Singletons	6
Mercaptopurine	Xanthine dehydrogenase/oxidase	Lymphatic Leukemia	Nucleobases	6
Pentostatin	Adenosine deaminase	Lymphoproliferative malignancies, particularly hairy-cell leukemia	Nucleobases	6
Pentoxifylline	Chitinase	Peripheral vascular diseases and cerebrovascular insufficiency.	Nucleobases	6
Pyridoxal Phosphate	D-serine dehydratase	Dietary shortage or imbalance.	Singletons	6
Quercetin	Xanthine dehydrogenase/oxidase	Dicrease capillary fragility.	Flavones	6
Telbivudine	Deoxycytidine kinase	HepatitisB virus (HBV)	Nucleobases	6
Triamterene	Pteridine reductase 1	Edema associated with congestive heart failure.	Aminopteridones	6

Listed are the drugs and their targets as well as their original indication and chemical class.

**Figure 7:**
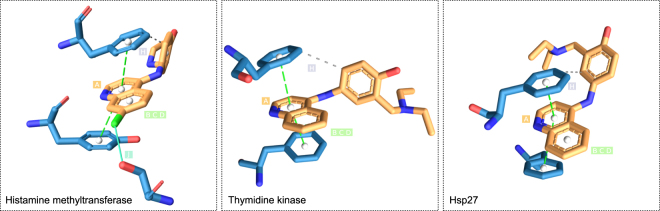
Amodiaquine in complex with its target histamine methyltransferase (2AOU, left), with the *in silico* docked target thymidine kinase (1OSN, middle) and Hsp27 model (right). All contain the patterns A–D and H.

Finally, we evaluated the potential of hit compounds to bind to BVDU’s primary target *Herpes simplex* thymidine kinase, the starting point for our pattern-based screening. Heinrich *et al*.^3^ showed that BVDU also binds the human heat shock protein, Hsp27^3^, and thereby blocks its anti-apoptotic activity allowing cytotoxic agents to reestablish their efficacy. To assess whether candidates could bind to these targets, we employed *in silico* docking with the widely used Autodock software (see Methods for details). We docked all 58 drugs to the relevant binding sites in the viral thymidine kinase and the a homology model of Hsp27^3^ and found that chrysin, olaparib, amodiaquine, and acenaphthenoquinone ranked better than BVDU in both cases (see Supplementary Fig. 1 for details). Figure 7 shows how the software docked amodiaquine to the two targets and how the docking poses satisfy the *π*-stacking patterns A–D as well as the distal hydrophobic contact pattern. However, they do not satisfy the hydrogen bond patterns E–G. In this respect, amodiaquine binds the BVDU targets in the same manner as one of its targets, histamine methyltransferase.

As a final step to test our structural repurposing approach with PLIP, we tested amodiaquine *in vitro* in biochemical and cell biological assays.

### Amodiaquine as anti-cancer drug?

**Figure 8:**
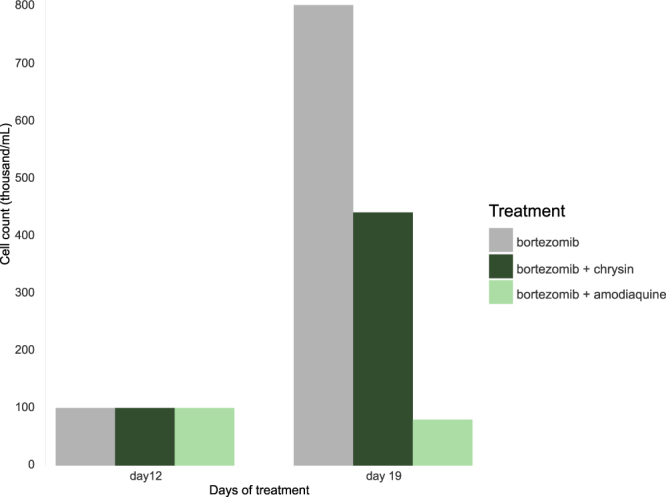
Drug-resistant multiple myeloma cells continue to grow despite treatment with the cytostatic drug bortezomib. However, when combined with bortezomib, amodiaquine significantly reduces cell numbers. Chrysin achieves a similar effect.

**Figure 9:**
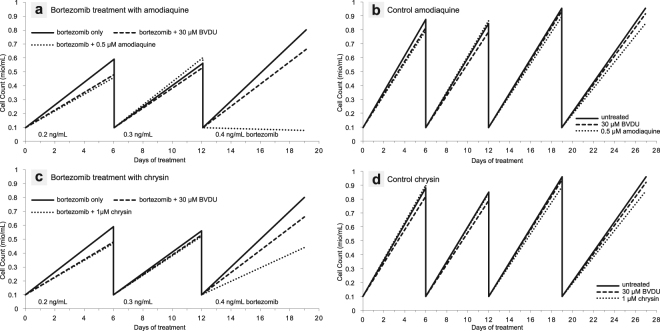
Detailed results of the cell resistance assay. (**a**) Amodiaquine re-establishes chemosensitivity in resistant cancer cells. Cells are treated with increasing doses of the cytotoxic agent bortezomib and amodiaquine over three passages. There is a clear difference in growth behaviour between treatment with bortezomib only or with addition of amodiaquine. In comparison to the known binder BVDU at 30, amodiaquine is more effective at 1/60th of this dose. (**b**) The control shows that amodiaquine is not a cytotoxic agent. When administered on its own (without bortezomib), there is no difference in cell growth compared to untreated cells. The same holds true for chrysin (**c**,**d**), although the effect on cell growth is not as strong. All experiments were conducted with the multiple myeloma cell line RPMI-8226.

BVDU exerts its anti-cancer effect by suppressing the cancer cells’ ability to develop resistance to a chemotherapy. Heinrich *et al*.^14^ demonstrated this by treating a multiple myeloma cell line with bortezomib, a chemotherapeutic agent, at increasing doses, which creates a selective pressure for the cells to develop resistance. Co-treatment with bortezomib and BVDU^14^ inhibits cell growth effectively and allows bortezomib to reestablish its cytotoxic effect. They also show that BVDU achieves this effect only in combination with bortezomib and has no cytotoxic activity on its own. We tested amodiaquine in the same chemoresistance assay to document its anti-cancer potential (Fig. 8). The cancer cells were exposed to increasing doses of bortezomib over three cell culture passages (see Fig. 9 for details). At the beginning of the third passage on day 12, 100,000 cells are exposed to the treatment regimes (bortezomib on its own and bortezomib with amodiaquine). After one week, resistance to bortezomib is clearly shown as cells continue to grow and have multiplied eightfold to 800,000. In contrast, the co-treatment with amodiaquine leads to a significant reduction in cell number to 80,000 (10%).

### Amodiaquine inhibits Hsp27’s chaperone function

In a biochemical assay, which elucidates how compounds inhibit the chaperone function of the heat shock protein Hsp27, Heinrich *et al*.^3^ showed that BVDU is a potent inhibitor. Straume *et al*.^15^ showed that breast cancer cells expressing Hsp27 become resistant to chemotherapy and that Hsp27 knockdown reestablished susceptibility. Furthermore, Bruey *et al*.^16^ showed that Hsp27 negatively regulates apoptosis via interaction with cytochrome C. Following this line of thought, Heinrich *et al*.^14^ devised an assay to show how BVDU impacts Hsp27’s function as a chaperone. Citrate synthase is used as a client protein of Hsp27 and misfolds at 43 °C, but in the presence of Hsp27 misfolding is inhibited. The chaperone activity of Hsp27 can be measured by the amount of misfolded client protein determined by capillary electrophoresis of precipitated protein. We tested amodiaquine in the Hsp27 chaperone assay and found that amodiaquine significantly inhibits Hsp27’s activity (see Methods for details) and that it was 43 more potent than BVDU. This activity is within the range of compounds tested in a previous study by Heinrich *et al*.^14^. Table 2 shows the results of the chaperone assay for the two tested compounds in comparison to lead compounds from the study by Heinrich *et al*.^14^.

**Table 2:** Results of the chaperone assay for amodiaquine, chrysin, and lead compounds from study by Heinrich *et al*.^14^ in comparison to the control BVDU.

	Compound	Dosage	Rel. CS Precip.	Rel. Inhib.
PLIP Screening	Chrysin	10 μM	0.92	69x
	Amodiaquine	10 μM	0.57	43x
Heinrich *et al*.	CAS 50-53-3	10 μM	1.06	80x
	CAS 61-00-7	10 μM	0.99	74x
	CAS 104715-80-2	10 μM	0.92	69x
	CAS 1222781-87-4	10 μM	0.81	61x
	CAS 161363-17-3	10 μM	0.71	53x
	CAS 1222812-38-5	10 μM	0.65	49x
	CAS 53-86-1	—	—	—
Control	BVDU	750 μM	1	1x

Listed are the dosage used in the experiment, the relative precipication of the client protein citrate synthase, and the relative inhibition after correcting for the dosage. Amodiaquine is substantially better than BVDU in inhibition of Hsp27 chaperone activity and within the activity range of compounds identified by Heinrich *et al*.^14^. The same is true for chrysin.

### Repositioning matrix

**Figure 10:**
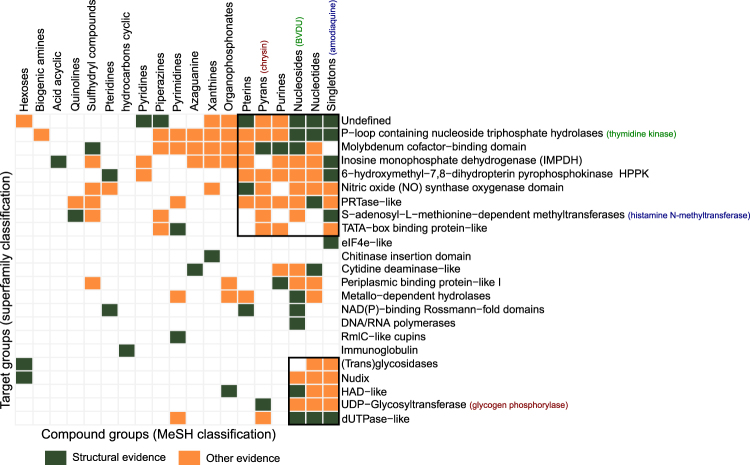
Evidence for binding of 58 drugs to 43 targets (grouped by scaffold type and superfamily). Green indicates structural evidence, orange other evidence. For 28% of pairs there is binding evidence.

Amodiaquine shares key interaction patterns with BVDU and also binds Hsp27. But to what extend is the ability to form these interaction patterns deterministic for target protein binding? Does amodiaquine also bind to the Herpes thymidine kinase and does BVDU bind to amodiaquine’s target histamine methyltransferase? In fact, do all of the 58 drugs with six or more BVDU interaction patterns bind to all of the 43 PDB targets? It is unlikely that this holds in its entirety, but it is reasonable to assume that some of these potential “repositioning cases” are valid. A systematic *in vitro* validation of all 58 drugs against all 43 targets was not carried out, however, a survey of the literature and databases supports many of these predicted interactions, and we grouped these drugs into 19 drug classes and the 43 targets into 23 superfamilies (see Methods). Figure 10 reveals whether there is evidence for target binding from crystal structures (green) or from other sources such as text mining, biological assays, or pathways (orange).

From all 437 possible binding combinations of proteins and compounds (Fig. 10), 41 (9%) could be confirmed by structural evidence (green). For 81 (18.5%) other drug-target pairings, there is evidence for binding from text mining, bioassays, screening or pathway data. Taken together, both sources provide binding evidence for 28% of all possible combinations. By arranging the data points by the number of shared targets (for compounds) and the number of shared ligands (for targets), clusters were produced in the matrix, which indicate that for some protein and ligand ensembles, evidence is available that they may bind to each other. One small distinct cluster (Fig. 10, lower right corner) is formed by dUTPase-like proteins, UDP-Glycosyltransferases, HAD-like protein, Nudix-, and (trans)glycosidases, together with nucleosides, nucleotides and singletons are supported by both structural and other evidence. A large cluster is visible in the top right of the matrix with 6 compound and 7 protein groups. Interestingly, this cluster also contains the chemical group of the hit compound amodiaquine and its target histamine methyltransferase (blue) as well as BVDU’s chemical group and the superfamily of BVDU’s target thymidine kinase (both marked in green font). The available evidence places the nucleosides as the compound group, which binds to the largest number of target superfamilies (17), while the P-loop containing nucleoside triphosphate hydrolases are known to bind to most (12) of the chemical classes from the set of 58 drugs.

### Validation on a different scaffold: chrysin

Out of interest, we took a closer look at the highest-ranking compound not in the list of FDA-approved drugs to experimentally confirm another binding prediction with a different chemical scaffold (Fig. 10). Evidence supporting the potential for binding of the flavone chrysin to Hsp27 is particularly strong: Chrysin’s interaction with rabbit glycogen phosphorylase (PDB ID 3EBO) satisfies the BVDU consensus patterns A (base ring), E (double hydrogen bonds), and H (distal hydrophobic contact), as well as the patterns B-D for *π*-stacking. It also was ranked best in the *in silico* docking experiment. Thus, we tested chrysin in the chemoresistance assay (see Methods) and Fig. 8 shows that, similar to amodiaquine, chrysin in combination with bortezomib reestablishes cytostatic activity and reduces cell growth by 50% in comparison to cells only with bortezomib alone. Growth of the drug-resistant cells is not completely stopped (as with amodiaquine), but is substantially reduced. In the Hsp27 chaperone assay, chrysin performs 69 times better than the reference compound BVDU (see Methods and Table 2).

## Discussion

The results we present in this work spark discussion on three points. Firstly, how does PLIP screening for drug repositioning compare to other computational approaches? Secondly, could the discovery of amodiaquine’s anti-cancer effect have been obtained differently? Thirdly, can the PLIP approach be applied to other indications?

PLIP utilizes interaction-based screening, in contrast to ligand- and target-based approaches, which are based on chemical or protein structure, respectively. Ligand-focused approaches^8–11, 17, 18^ usually consider molecular structure properties or fragment composition to find similar compounds. This is successful if the goal is the discovery of structurally related compounds. However, to identify novel scaffolds (ligand hopping), this method is not suitable. In our example, amodiaquine is structurally unrelated to BVDU and could therefore not have been found by these methods. Target-focused methods^6, 7, 19–23^ rely on either geometrical analysis or chemical analysis of the target protein. Similar to ligand hopping, these methods do not lend themselves to target hopping. As an example, BVDU binds a thymidine kinase and amodiaquine a methyltransferase. These targets do not share any similarities, however, by focussing on the drug-protein interactions, PLIP can find a relationship between BVDU/thymidine kinase and amodiaquine/methyltransferase, since they interact in a very similar manner. This focus on interactions rather than on ligands or target is also implemented in other tools^24, 25^, however, these do not consider some interaction types (*π*-stacking and/or halogen bonds), which were necessary to define the BVDU patterns and finally to make the link to amodiaquine and other hit compounds.

Based on drug-target interaction patterns, PLIP identified the anti-cancer potential of the anti-malaria drug amodiaquine. We verified this anti-cancer effect by showing that multiple myeloma cancer cells, which have developed resistance to chemotherapy, become sensitized to chemotherapy again upon exposure to amodiaquine. One important question here is whether amodiaquine is also a cytotoxic agent or works via a specific mechanism on cell resistance. The resistance assay clearly shows that amodiaquine has a specific and synergistic effect with the cytotoxic agent bortezomib (see Fig. 9). Without the cytotoxic agent (control runs a + b), the cells can grow unhindered, so a cell toxicity of amodiaquine can be excluded. When administered in combination with bortezomib, however, the growth behaviour clearly changes due to a re-establishing of chemosensitivity by amodiaquine. The same is true for chrysin, although the effect on cell growth is not as strong.

We show that amodiaquine inhibits the chaperone function of the heat shock protein Hsp27, which plays a key role in the chemoresistance of some tumor types^15^. These findings are supported by Qiao *et al*.^26^, who treated cultured malignant melanoma cells with amodiaquine and found that it sensitized them to starvation- and chemotherapeutic-induced death. They also showed that heat shock proteins, Hsp70 and Hsp90, play an important role in this process, similar to Hsp27 in our studies.

Unlike most repositioned drugs, Qiao *et al*.^26^ used a rational approach to identify amodiaquine. The authors argued that autophagy and lysosomal degredation play an important role in cancer^27, 28^ and that, as many anti-malaria drugs are lysosomotropic, they set out to screen anti-malarial agents for their anti-cancer behaviour. Their approach to drug repositioning is nonetheless serendipitous, since the hypothesis was not generated systematically. However, it also shows how techniques such as text-mining, reasoning, mechanism of action insights, and database integration will play an important role in the systematization of drug repositioning.

In contrast to Qiao *et al*.^26^, PLIP’s identification of amodiaqine was based on an algorithmic processing of data on its target, methyltransferase, which shares its binding site interactions with BVDU’s targets Hsp27 and thymidine kinase. We obtained proof that amodiaquine inhibits Hsp27 function, but binding to thymidine kinase and any anti-viral activities were not tested. However, there is evidence that amodiaquine inhibits virus replication of flaviviridae including dengue virus^29^ and there is also evidence that it interferes with thymidine production^30^. Such circumstantial evidence supports future experiments to test amodiaquine’s anti-herpetic activity.

PLIP’s ranking of compounds by the number of matching interaction patterns was crucial and very efficient: 13,000 compounds in the PDB are reduced to just 247 compounds containing at least six BVDU patterns, including a mere 12 FDA-approved drugs, one being amodiaquine. This drug shows a double *π*-stacking sandwich, which is highly specific (0.5% of interactions in PDB), a hydrophobic contact and a halogen bond, but is missing the parallel hydrogen bonds. However, the latter is a consensus interaction pattern among the five structures (Fig. 3), which means that a search for this consensus would not have included amodiaquine. Thus, we believe, that a ranking by number of patterns is more advantageous than filtering by a defined set of patterns. Ranking by number of patterns becomes even more powerful if combined with a chemical similarity analysis to exclude structurally obvious hits and focus on compounds with novel scaffolds. In our study, 58 hit compounds (Fig. 4) contain 40 nucleobases, which are chemically similar and hence not novel, leaving only 18 compounds with a good match in interaction patterns, but highly dissimilar to BVDU and hence, are novel scaffolds.

In our previous work (Heinrich *et al*.^14^) we identified novel Hsp27 inhibitors by target hopping from thymidine kinase to Hsp27. We argued that if one thymidine kinase binder (BVDU) inhibits Hsp27, then so may others, and identified 249 thymidine kinase binders, that were reduced to 29 with better *in silico* binding affinity than BVDU. Six of these 29 were validated *in vitro* in the same chemoresistance and chaperone function assays used here for amodiaquine. Both approaches largely complement each other, since in our earlier work, we did not require structural data to identify the 249 compounds. Hence, there is only a small overlap of 10 compounds with our candidates. However, the success of our work^14^ means that all of the 43 targets we identified with PLIP can serve as a starting point to collect binders, which could then be ranked and experimentally validated.

Next to amodiaquine from our FDA-approved hit candidates, we selected chrysin, a natural compound, as a second candidate for validation, since the interaction pattern score from the PLIP screen was high. Result from the *in vitro* chemoresistance and chaperone assays were good, although not as potent as amodiaquine. Chrysin has shown beneficial effects in cancer cell lines^31–34^ and additionally, a moderate antiviral activity was demonstrated against *H*. *simplex*, *V*. *zoster*, and human Enterovirus 71^35–39^. These findings are consistent with the hypothesis that chrysin binds Hsp27 and the herpes thymidine kinase.

However, chrysin also has anti-allergic and anti-inflammatory activity^33^ and contains a catechol group with promiscuous binding behaviour^40^. Many potential targets have been proposed^34^ and there is also structural evidence that its binding can be very different from the patterns discussed above. For example, chrysin binds to the transport protein transthyretin (PDB ID 4DES) with low affinity^41^ using almost exclusively hydrophobic contacts and just one hydrogen bond to a lysine at the entrance of the binding site. The *in vitro* effects of chrysin require very high micromolar concentrations^42–44^, so while chrysin is an interesting compound, whose binding behaviour supports our structural approach to drug repositioning, in contrast to amodiaquine, chrysin’s pharmacological profile does not support further development as an anti-cancer lead substance.

To estimate how widely applicable PLIP screening is, consider the Repurposed Drug Database^45^ as a reference. This lists 233 repositioned drugs and for 56 of these (24%), there is structural data for the ligand and one therapeutic target. This means that for 24% of the repurposed drugs, there is similar coverage of structural data as there was for our case using BVDU patterns. Similarly, there is structural data for more than 25% of FDA-approved drugs (400 out of ca. 1600^46^). Hence, while there is far less structural data than, e.g. sequence data, there is nonetheless a substantial amount, which is sufficient for structural approaches, such as PLIP, to be a viable tool beyond examples presented in this paper.

Concluding, we could show that our structure-based screening approach with PLIP interaction patterns identifies novel repositioning candidates for the cancer target Hsp27. Not only did we identify candidates structurally unrelated to the query drug BVDU (scaffold hopping), but also demonstrated that they show the desired inhibitory activity on the target protein Hsp27 and suppress chemoresistance. Especially the FDA-approved malaria drug amodiaquine, which emerged as the top hit from our screen and was subsequently validated, proves the potential of our approach for drug repositioning.

## Methods

### Interaction patterns

107,663 structures were downloaded from the Protein Data Bank (PDB)^47^ FTP Archive on Apr 2015. Each structure was analysed with PLIP v1.1.1^12^ using standard settings, resulting in 408,877 complexes. These were filtered using BioLiP, a database for discrimination between biologically relevant ligand and artifacts^48^, to keep 170,219 complexes.

To construct BVDU interaction patterns, the PDB structures 2W0S, 2JAW, 1OSN, 2VQS, 1KI8 (which contain BVDU) were analysed with PLIP, resulting in fourteen complexes overall. The structure 4XSC was not available at that time (released date Dec 2016), but his absence does not imply any significant change in the analysis. Pattern definitions include one or two specific non-covalent interactions and their relative orientation (angle or distance ranges). The ten pattern are shown in Fig. 2 and their definitions listed in Table 3.

**Table 3:** Definitions of patterns A–J.

	Pattern	Geometric Constraints
A	Base pattern: aromatic ring in ligand	
B	Double *π*-stacking to base ring	
C	Double *π*-stacking to base ring on opposite sites	
D	Double *π*-stacking to base ring in parallel type	
E	Double hydrogen bonds to base ring	
F	Double hydrogen bonds to shared residue	
G	Double hydrogen bonds in paralell	angle is 180° ± 18°
H	Distal hydrophobic contact	dist. to ring 4.0 Å < x < 6.5 Å
I	Distal hydrogen bond	dist. to ring 5.4 Å < x < 6.1 Å
J	Distal halogen bond	dist. to ring 7.8 Å < x < 9.2 Å

The base pattern A is required in every complex. For the patterns G to J, additional geometric constraints for their definition were necessary and have been derived from the observed geometries in BVDU(−MP) complexes.

The 170,219 complexes were screened for the presence of the ten patterns and if a ligand was represented by several complexes, the complex with the most BVDU patterns was selected. Figure 4 shows the distribution of pattern numbers for these complexes. Of these complexes, 247 have six or more patterns and 58 of these are drugs, classified as experimental, clinical, or approved drugs based on the Therapeutic Target Database (TTD)^49^.

### Chemical similarity

To prioritize the 58 compounds they were manually assigned to scaffold types and their pairwise chemical similarity was computed using the PubChem Score Matrix Service (pubchem.ncbi.nlm.nih.gov/score_matrix) with standard settings. Chemicals were downloaded from PubChem in SDF format. A heatmap of the pairwise scores was generated with the Heatplus package v2.16.0 in R 3.2.5. Compounds on the axes were grouped by scaffold and within each scaffold by hierarchical clustering on average distances.

### Docking

Docking was performed with AutoDock 4.2 rigid body docking on the known BVDU binding site in 1OSN and in the model of Hsp27, as described by Heinrich *et al*.^3^. Structures were prepared with AutoDockTools v1.5.4 to assign atom types and partial charges. The docking area was defined in the respective binding pockets by a box of 60 × 60 × 60 Å for 1OSN and 50 × 60 × 50 Å for Hsp27 with 0.375 Å spacing. Lamarckian genetic algorithm with 150 randomly placed entities, 27,000 generations, 5,000,000 energy evaluations, a mutation rate of 0.02, elitism value 1, and a cross-over rate of 0.80 was used for the docking process, with a total of thirty runs per compound. The local search was performed using the Soils and Wets algorithm with 300 iterations per search. After the docking, pose clusters were generated using Root Mean Square Deviation (RMSD) values.

### Chemoresistance assay

BVDU was a gift from Rudolf Fahrig, RESprotect GmbH, Dresden (Germany). Amodiaquine and chrysin were bought from Sigma-Aldrich. A multiple myeloma cell line (RPMI-8226) was obtained from the DSMZ (German Collection of Microorganisms and Cell Cultures). The cells were cultivated in RPMI 1640 medium, supplemented with 10% (v/v) fetal bovine serum in a humidified atmosphere at 37 °C and 5% *CO*_2_. Cells in logarithmic growth phase were reseeded at a density of 100,000 cells per mL and incubated with the chemotherapeutic agent bortezomib (Velcade) together with the test compounds. Bortezomib was omitted for the control run. Cells were passaged regularly to prevent densities of more than 1,000,000 cells per mL. Bortezomib was added at an initial dose of 0.1 ng/mL, followed by increasing doses of 0.2 ng/mL (second passage) and 0.3 ng/mL (3rd passage). As a positive control, cells were incubated with bortezomib and 30 μM BVDU. For all tested compounds, non-toxic doses were previously determined.

### Hsp27 chaperone function assay

Hsp27 is a chaperone for many client proteins including citrate synthase (CS). CS misfolds at temperatures above 43 °C, but in the presence of Hsp27, misfolding is reduced. Inhibitors binding to Hsp27 impair its chaperone function, leading to accumulation of denatured CS and the amount of misfolded protein can thus serve as a measure of Hsp27 inhibition. BVDU, controls, or test compounds were incubated in 40 mmol L-HEPES buffer with 1.44 μM CS and 481 nM Hsp27 at 43 °C and pH 7.4. Samples were taken after 30, 60 and 90 min and aggregated CS quantified using capillary electrophoresis. The relative inhibitory values were calculated by measuring the amount of misfolded CS and subtracting the value of the blank (DMSO buffer) and all values were normalized to the value for BVDU and the concentration of test compounds used.

### Repositioning matrix

We constructed a “repositioning matrix” of the 58 compounds, satisfying six or more patterns, and their 43 targets. For each possible compound-target pair we reviewed the databases STITCH, Chembl, and PDB for binding evidence. STITCH v5.0^50^ was accessed in Feb 2017, ChEMBL^51^ in Aug. 2016 and PDB^47^ in Apr. 2015. STITCH target IDs were mapped through the file full_uniprot_2_string.04_2015.tsv.gz. ChEMBL compounds were mapped by PubChem, ChEMBL targets by name.

Since the data points in the repositioning matrix are very scattered, we aggregated data by mapping target domains to their respective superfamilies using SCOPe^52^ (accessed in Oct 2016). This reduced the 43 targets to 23 superfamilies. The 58 compounds were mapped to MeSH chemicals and drugs categories, as available from PubChem (accessed in Oct 2016), which reduced 58 compounds to 19 classes.

### Data availability

All primary data used for the analysis stems from public sources, which are described in the methods section of the paper. If required, identifiers are provided in the corresponding sections. PDB identifiers for the screening set as well as detailed information on hit compounds and their PubChem Compound Identifiers (CIDs) are provided as Supplementary Information. Source data for Fig. 5 is provided as Supplementary Information. All other relevant data are available from the corresponding author on request.

### Code availability

The Protein-Ligand Interaction Profiler (PLIP) as the key software for the study is publicly available at github.com/ssalentin/plip under the open source Apache 2 license.

## Electronic supplementary material

Supplementary Information

Supplementary Dataset 1A

Supplementary Dataset 1B

Supplementary Dataset 1C

Supplementary Table 1
