# Supplementary material for: From malaria to cancer: Computational drug repositioning of amodiaquine using PLIP interaction patterns
Source: Sci Rep. 2017 Sep 12;7:11401. doi: 10.1038/s41598-017-11924-4 (PMC5595859; doi:10.1038/s41598-017-11924-4)
Supplement: Supplementary file 1 — Supplementary Information [file 41598_2017_11924_MOESM1_ESM.pdf]

From malaria to cancer: Computational drug repositioning of amodiaquine using PLIP interaction patterns

Sebastian Salentin[1†], Melissa F. Adasme[1†], Jörg C. Heinrich[1], V. Joachim Haupt[1], Simone Daminelli[1], Yixin Zhang[2] & Michael Schroeder[1*]

1 Biotechnology Center (BIOTEC), Technische Universität Dresden, 01307 Dresden, Germany

2 B CUBE, Center for Molecular Bioengineering, Technische Universität Dresden, 01307 Dresden, Germany

* ms@biotec.tu-dresden.de

†these authors contributed equally to this work

Data

Three files with raw data from intermediate steps of the analysis workflow are provided as supplementary data:

- **S1_dataset1.tds. Screening dataset.** Dataset of 107,663 complexes used for virtual screening of BVDU interaction patterns. Listed are the PDB ID, HET ID of the ligand, its assigned chain and position in the chain.
- **S1_dataset2.tds. Non-redundant dataset after screening.** Non-redundant dataset of 15,712 complexes after virtual screening. Listed are the PDB ID, HET ID of the ligand, its assigned chain and position in the chain.
- **S1_chemsim.tds. Pairwise chemical similarity.** Pairwise chemical similarity values of the 58 compounds in the candidate set. The given values are Tanimoto similarities between the standard PubChem chemical fingerprint (between 0 and 100 % similarity). Compounds are reference with their PubChem CID.

Supplementary Table 1 is supplied as a separate Excel spreadsheet (SI_candidate_compounds.xlsx).

Figures

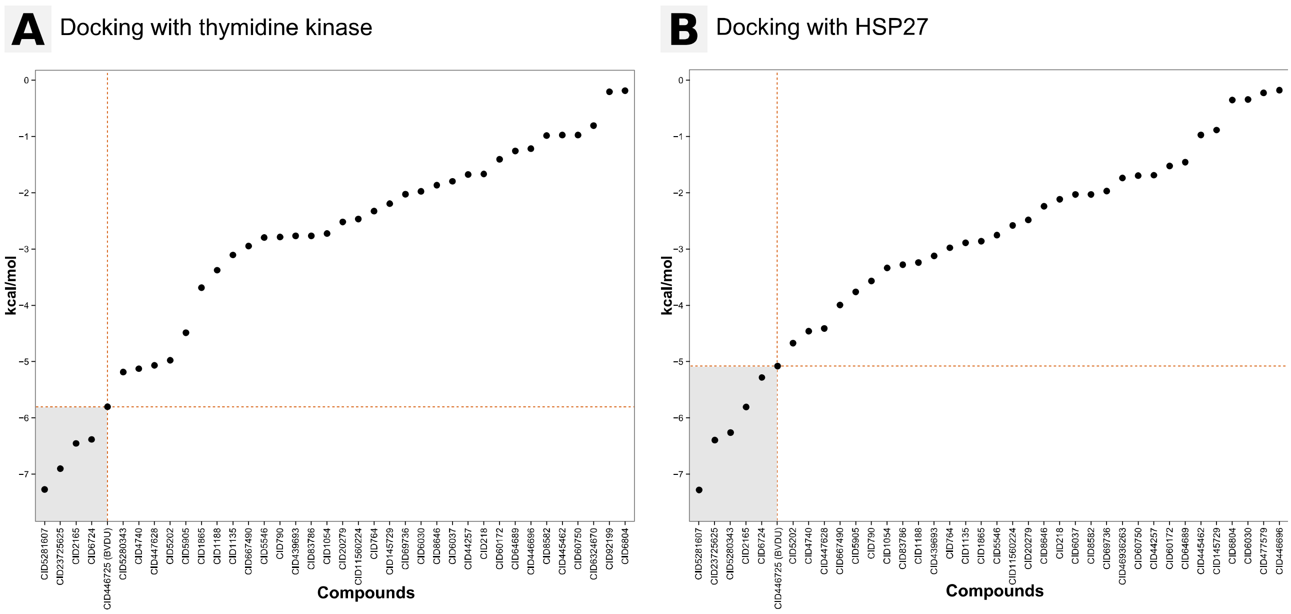

Supplementary Figure 1. Docking Results for candidate compounds to viral thymidine kinase (left) and the Hsp27 model (right). Predicted binding energies are shown on the ordinate in kcal/mol. Compounds are listed on the abscissa with their PubChem CIDs. BVDU was used as a reference compound and its data points are indicated with dotted lines.

Tables

Supplementary Table 1. Candidate compounds after virtual screening. Detailed information is listed for all compounds having 6 or more matching BVDU interaction patterns (candidate set). Listed for each compound are its PubChem Compound ID (CID), the name, its status as a drug (approved, clinical, or experimental), the chemical class as described in the manuscript, and the number of matched BVDU patterns.
